# Novel Therapeutic Strategies for Squamous Cell Carcinoma of the Head and Neck: Beyond EGFR and Checkpoint Blockade

**DOI:** 10.3390/biomedicines13081972

**Published:** 2025-08-14

**Authors:** Rachel Hui Zhen Sim, Pei Jye Voon, Seng Wee Cheo, Darren Wan-Teck Lim

**Affiliations:** 1 Division of Medical Oncology, National Cancer Centre Singapore, Singapore 168583, Singapore; darren.lim.w.t@singhealth.com.sg; 2 Department of Radiotherapy, Oncology and Palliative Care, Hospital Umum Sarawak (Sarawak General Hospital), Kuching 93586, Malaysia; voonpj@yahoo.com; 3 Department of Radiotherapy and Oncology, Hospital Wanita Dan Kanak-Kanak Sabah, Kota Kinabalu 88996, Malaysia; cheosengwee@gmail.com; 4 Centre for Clinician Scientist Development, SingHealth Duke-NUS, Singapore 169857, Singapore

**Keywords:** head and neck cancer, squamous cell carcinoma, novel therapies, targeted therapy, immunotherapy, epigenetics, tumor microenvironment, cell therapy, oncolytic virus, drug resistance

## Abstract

Despite advances in immunotherapy with checkpoint inhibitors, a significant proportion of patients with head and neck squamous cell carcinoma (HNSCC) do not respond to treatment or eventually develop resistance. This review focuses on novel therapeutic strategies currently under investigation for HNSCC, moving beyond the established paradigms of EGFR inhibition and PD-1/PD-L1 blockade. We explore emerging targets and drug classes, including next-generation immunotherapies, targeted therapies directed at specific molecular alterations, epigenetic modifiers, agents targeting the tumor microenvironment, and innovative approaches like cell-based therapies and oncolytic viruses. We discuss the preclinical rationale and clinical data (where available) for these novel approaches, highlighting the challenges and opportunities in translating these discoveries into improved outcomes for patients with HNSCC.

## 1. Introduction

Globally, head and neck cancers account for approximately 826,000 cases and 408,000 deaths annually [1]. These cancers include neoplasms of the oral cavity, oropharynx, hypopharynx and larynx and are most commonly squamous cell carcinoma histology. Risk factors commonly associated with squamous cell carcinoma of the head and neck (HNSCC) include smoking, human papillomavirus infection (HPV) and alcohol use.

Patients often present with locally advanced disease warranting a multi-disciplinary approach to treatment, entailing surgical resection (if deemed resectable) followed by adjuvant therapies like radiotherapy and chemotherapy; whereas those with unresectable disease are traditionally offered concurrent chemoradiation. Due to the heterogenous nature of HNSCC as well as its associated morbidity, treatment is often challenging requiring multimodality approaches and recurrence rates remain high in spite of these aggressive therapies rendered [2].

HNSCC remains a challenging malignancy to treat, especially in the recurrent or metastatic (R/M) setting where treatment options are limited. While epidermal growth factor receptor (EGFR) inhibitors (e.g., cetuximab) and PD-1/PD-L1 checkpoint inhibitors (e.g., pembrolizumab, nivolumab) have improved outcomes for some patients, many do not have a sustained response and eventually develop resistance [3,4]. This necessitates the development of newer therapeutic approaches that can allow us to target alternative pathways to overcome drug resistance. This review will focus on promising novel approaches currently under investigation for HNSCC.

## 2. Next-Generation Immunotherapies

HNSCC presents a unique immune landscape that makes it an attractive target for immune-driven therapeutic approaches. This is especially evident in the context of HPV-positive tumors, which are regarded as immunogenically ‘hot’ tumors rich with high immune cell infiltration. HPV-negative tumors, however, have a poorer prognosis as compared to their HPV-positive counterparts as they tend to be immunologically ‘cold’. In spite of this, these tumors are enriched in neoantigens related to smoking exposure and are also associated with higher mutational burdens [5].

This demonstrates that HNSCC has the potential to derive significant clinical benefit from an immunotherapy-based approach, resulting in a paradigm shift in treatment options with greater emphasis on incorporation of immune-driven therapeutic agents rather than traditional cytotoxic therapy in emerging trials.

Pembrolizumab with or without chemotherapy, has now become the standard of care in first line R/M HNSCC based off the results of KEYNOTE 048, the pivotal study that demonstrated superiority of Pembrolizumab over Cetuximab with chemotherapy in patients with a combined positive score (CPS) of ≥1 [3].

While PD-1/PD-L1 blockade has become a cornerstone of HNSCC treatment, research is actively exploring ways to enhance immunotherapy efficacy as the overall survival (OS) of these patients still remains poor.

### 2.1. Targeting Alternative Immune Checkpoints

As a result, there has been growing interest in attempts to augment the PD-1/PD-L1 blockade or target alternative checkpoints in a bid to further improve on outcomes for our refractory or metastatic HNSCC patients.

Cytotoxic T lymphocyte-associated antigen 4 (CTLA-4) antibodies have unfortunately shown limited efficacy as monotherapy in HNSCC. However, other combinations with PD-1 inhibitors are being explored, though with limited success thus far with CHECKMATE 651 demonstrating no survival benefit of Ipilimumab Nivolumab over Cetuximab chemotherapy [6].

Lymphocyte-activation gene 3 (LAG-3) is another inhibitory receptor expressed on T cells, thus emerging as a new potential target in the field of inhibitory checkpoints. LAG-3 is known to have an inhibitory effect by downregulating T cell receptor signalling, thus resulting in reduced immune activation [7]. In HNSCC, LAG-3 overexpression has also been associated with a poorer prognosis, typically resulting in larger sized tumors, more extensive nodal involvement and a higher pathological grade [8]. LAG-3 inhibitors like Favezelimab are currently being investigated in combination with PD-1 blockade in advanced solid cancers [9]. In HNSCC, these drugs have demonstrated efficacy in combination with immune checkpoint inhibitors—Fianlimab with Cemiplimab as well as Eftilagimod Alpha with Pembrolizumab have demonstrated response rates of up to 30% or more [10,11].

T-cell immunoglobulin and mucin-domain containing-3 (TIM-3) is an immune checkpoint expressed on T cells and other immune cells. TIM-3 inhibitors are in early clinical development with preliminary studies in mice demonstrating that when TIM-3 inhibitors are used in combination with radiotherapy and PDL-1 inhibitors, this resulted in cessation of tumor growth, though the effects were short lived [12].

T cell immunoreceptor with Ig and ITIM domains (TGIT) are inhibitory receptors expressed on T cells and NK cells. Anti-TIGIT antibodies are being tested in combination with PD-1/PD-L1 inhibitors in mice models, with the combined blockade of TIGIT and PD-L1 signaling resulting in reduced tumor growth, increased effector T cells and subsequently enhanced immune memory effects [13].

### 2.2. Bispecific Antibodies

Bispecific antibodies contain two distinct binding domains that are engineered to have the unique ability to attach to two targets simultaneously, thus enabling different mechanisms of action that were not previously achievable with the use of traditional IgG-based antibodies. This can enhance T cell engagement and tumor cell killing.

Several bispecific antibodies are in development for HNSCC, including those targeting EGFR and CD3 (to recruit T cells to EGFR-expressing tumor cells) [14]. HNSCC tumors are associated with high expression of EGFR in up to 80–90% of patients, some of the highest rates amongst all cancers [15]. Additionally, overexpression of cMET, a proto-oncogene, has been associated with worsened prognosis and inferior survival [16], with some proposing that it may even provide a mode of resistance against anti-EGFR treatments due to its involvement of similar pathways in the PI3K/Akt and MAPK nodes [17]. This provides an appropriate avenue for bispecific molecules to come into play. Amivantamab targets both EGFR and MET and is currently being studied in HNSCC. Early positive results were seen in a HNSCC patient whose EGFR and MET expressing tumor was transplanted into humanized patient-derived xenograft models and exposed to Pembrolizumab with Amivantamab. Significant reduction in tumor size was observed along with increased CD8+ T cell activity that contributed to creating a more favourable tumor microenvironment (TME) [18].

Another bifunctional antibody targeting EGFR and transforming growth factor-beta (TGF-B) called BCA101 showed a clear signal of efficacy in treatment naïve HNSCC when given in combination with Pembrolizumab [19]. This dual blockade was hypothesized to have synergistic anti-cancer effect by disrupting the epithelial–mesenchymal transition whilst enhancing natural killer cell activity and antibody-dependent cellular cytotoxicity. Objective response rates (ORR) were as high as 46% with a significantly better response in the HPV-positive subgroup.

Petosemtamab targets both EGFR as well as leucine-rich repeat-containing G-protein coupled receptor 5 (LGR-5), which is known to be upregulated in cancer and is also pro-oncogenic by encouraging cell proliferation and reducing apoptosis. This bispecific antibody has been demonstrated to be efficacious when given together with Pembrolizumab, with a remarkable ORR of 67% in treatment of naïve R/M HNSCC [20].

Human epidermal growth factor receptor 3 (HER3) is overexpressed in HNSCC and portends a poorer prognosis [21]. It has been described in the literature to be a possible mechanism for resistance to anti-EGFR inhibitors such as Cetuximab as it causes dysregulation of EGFR degradation. This makes HER3 inhibitors an attractive and promising target for HNSCC [22]. Izalontamab targets both EGFR and HER3 and was shown to have an ORR of up to 64.3% when combined with taxanes in R/M HNSCC patients who had progressed on prior immunotherapy but were naïve to platinum-based chemotherapies [23].

### 2.3. Chimeric-Antigen Receptor (CAR) T-Cell Therapy

Chimeric antigen receptor (CAR) T-cell therapy involves engineering a patient’s T cells to express a CAR that recognizes a specific tumor antigen. While CAR T-cell therapy has shown remarkable success in hematologic malignancies, its application in solid tumors, including HNSCC, is more challenging. Targets that are currently being explored for CAR T-cell therapy in HNSCC include EGFR and HER2, amongst others [24].

As previously mentioned, EGFR expression is common in HNSCC and CAR-T cells targeting this receptor have demonstrated significant tumor control in EGFR positive HNSCC cell lines in early trials, as a result of enhanced cytokine production and improved target cell death after co-culture of CAR-T cells with target cells [25].

Together with EGFR, HER2 has been found to be co-expressed in HNSCC and as previously mentioned, some studies suggest that this may contribute to resistance to anti-EGFR agents [26]. HER2 can be detected in up to 47% of HNSCC patients and is associated with a worse prognosis [27], thus making it an attractive targeted for CAR-T cell therapy—HER2 targeting CAR-T cells have demonstrated good activity in early studies, with a decrease in tumor size of up to 56% in chickens engrafted with HER2-positive HNSCC cell lines [28].

As of current time in publishing, CAR T-therapy is still being investigated in the setting of HNSCC, with many of the trials still in preclinical phase as translation into clinical research in human trials has proven to be challenging due to issues like physical blockades formed by the heterogenous tumor microenvironment (TME) of solid tumors, physiochemical barriers relating to the downregulation of cytokines and the immunosuppressive environment of solid cancers overall that restrict the access of CAR-T cells into these tissues [29].

### 2.4. Cytokine-Based Therapies

Interleukins (ILs) and other cytokines can modulate the immune response. Recombinant IL-2 has been used in cancer therapy for many years, but its toxicity remains a major limitation. Novel approaches include engineered cytokines with improved properties and cytokine-based immunotherapies. IRX2 has been explored in the setting of recurrent or metastatic HNSCC—this multi-cytokine immune-activating agent has demonstrated anti-tumor activity in a Phase IB trial in patients with refractory or metastatic HNSCC. When given together with Durvalumab, it demonstrated a modest response rate of 5.3% with disease control rates of 42% [30] with no dose-limiting or Grade 3 or 4 adverse reactions observed.

This same drug is now being tested in earlier stage HNSCC patients, with its neoadjuvant delivery prior to surgery demonstrating a significant increase in tumor infiltrating lymphocytes on pathological specimens after resection, indicating that IRX-2 is able to trigger a significant immune response in HNSCC [31].

### 2.5. Oncolytic Viruses

Oncolytic viruses are designed to selectively replicate and lyse tumoral cells, with minimal impact in normal tissues. They are also able to promote immunogenic cell death and stimulate an anti-tumour immune response [29].

An oncoloytic herpes simplex type-1 (HSV-1) virus was conditioned to trigger oncolysis of infected tumor cells and activation of the immune system via virus-mediated human granulocyle/macrophage colony stimulating factor expression and tumor-associated antigen release. Intra-tumoral injection of this oncolytic virus with chemoradiotherapy followed by surgical resection in locally advanced HNSCC patients resulted in response rates of approximately 82.3% and pathological complete response in 93% of patients in a Phase I/II trial [32].

**Figure 1:**
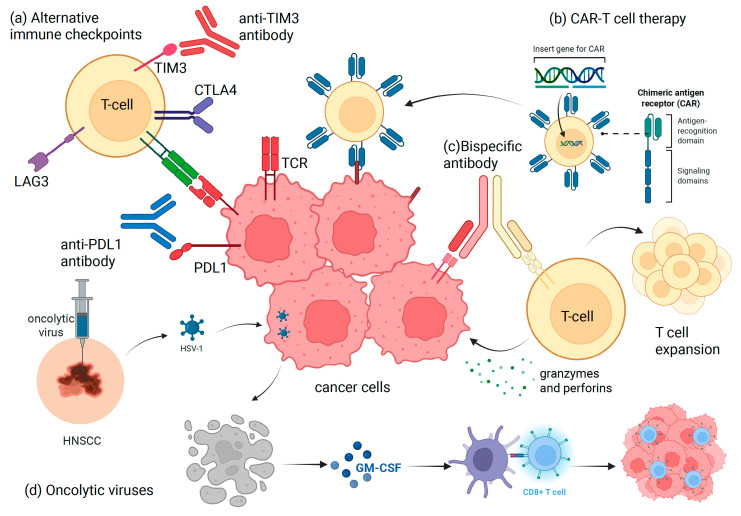
Graphical depiction of some of the immune-based novel approaches in HNSCC for consideration. (**a**) Antibodies targeting alternative immune checkpoints such as TIM-3, CTLA-4, LAG-3. (**b**) CAR-T cell therapy engineered to recognize specific HNSCC molecular targets. (**c**) Bispecific antibodies that can target two simultaneous binding domains to enhance T cell efficiency and engagement. (**d**) Oncolytic viruses targeting HNSCC-related viruses like HSV-1, activating CD8+ T cells through virus-mediated GM-CSF release. CAR, chimeric antigen receptor; CTLA-4, Cytotoxic T lymphocyte-associated antigen 4; HNSCC, head and neck squamous cell carcinoma; GM-CSF, granulocyte/macrophage colony stimulating factor; HSV-1, Herpes Simplex Virus-1; LAG-3, Lymphocyte-activation gene 3; PDL-1, programmed death-ligand 1; TCR, T cell.

Figure 1 summarizes some of the different novel immune-based approaches that we can consider other than the conventional PDL-1 blockade. This includes targeting alternative immune checkpoints (TIM3, CTLA-4, LAG-3), engineering therapies such as CAR-T or bispecific antibodies or designing oncolytic viruses to target specific HNSCC-related viruses.

## 3. Targeted Therapies

### 3.1. EGFR Mutations

HNSCC tumors are associated with high expression of EGFR in up to 80–90% of patients, some of the highest rates amongst all cancers [15]. Cetuximab (anti-EGFR monoclonal antibody) is thus far the only FDA approved drug available for use in relapsed or metastatic HNSCC—however it provides a low ORR of approximately 10% [33], likely due to multiple complex signaling pathways responsible for tumorigenesis besides EGFR, as well as high rates of acquired resistance [34]. EGFR tyrosine kinase inhibitors (TKIs) like Gefitinib have been extensively studied in HNSCC, but again have resulted in low response rates of 11% and, thus, none have been approved by FDA as of 2024 [35].

The challenge with anti EGFR-directed treatments is the inevitable development of acquired resistance—commonly through secondary mutations in the EGFR gene or the emergence of alternative activating signaling pathways or resistance to anti-apoptosis mechanisms [36]. Furthermore, the activation of the EGFR pathway triggers a multitude of diverse intra-cellular responses, hence inhibition of the EGFR alone is inadequate to control activation of its downstream signaling pathways, resulting in the inevitable progression of HNSCC.

In view of these challenges, other molecular alterations are now being explored and appear to offer additional opportunities for targeted therapy in HNSCC.

### 3.2. PI3K/AKT/mTOR Pathway

**Figure 2:**
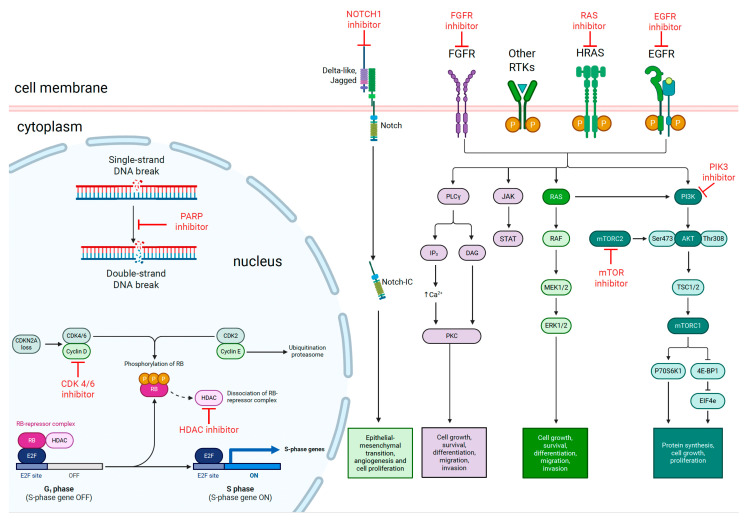
Depiction of how targeted therapies and epigenetic modifiers can provide an anti-cancer effect. Akt, Protein kinase B; CDKN2A, cyclin D1 cyclin-dependent kinase inhibitor 2A; Rb, retinoblastoma protein; EGFR, epidermal growth factor receptor; E2F, E2 transcription factor; FGFR, fibroblast growth factor receptor; HDAC, histone deacetylase; P, phosphorylation; PARP, poly ADP ribose polymerase; PI3K, Phosphatidylinositol 3-kinase; mTOR, mammalian target of rapamycin; Rb, retinoblastoma; RAS, rat sarcoma oncogene; RTK, receptor tyrosine kinase; S6K1, ribosomal protein S6 kinase 1; 4E-BP, 4E-Binding Protein 1.

Activating Phosphatidylinositol 3-kinase catalytic subunit alpha (PI3KCA) mutations result in uncontrolled cell proliferation and anti-apoptosis mechanisms via upregulation of the protein kinase B-mammalian target of rapamycin (Akt-mTOR) pathway. This pathway serves to activate mTOR Complex 1 (mTORC1) and its downstream molecules of ribosomal S6 kinase (S6K) and eukaryotic translation initiation factor 4E binding protein 1 (4E-BP1), ultimately resulting in mRNA translation and protein synthesis as depicted in Figure 2. PI3K mutations can be found in up to 30% of HNSCC tumors and, thus, PI3K inhibitors may prove to be useful in halting this cycle of excessive cell proliferation [37].

While pan-PI3K inhibitors have shown limited efficacy and significant toxicity, selective PI3K inhibitors (e.g., alpha-specific inhibitors like Alpelisib) and dual PI3K/mTOR inhibitors are being investigated [38]. A phase 2 study looked at recurrent/metastatic platinum refractory HNSCC patients naive to anti-EGFR therapy who were given Cetuximab alone vs. Cetuximab with Alpelisib. ORR was marginally better with combination therapy (9.9% vs. 5.7%) though with no difference in progression free survival (PFS) or OS. Overall, the addition of Alpelisib to Cetuximab unfortunately did not demonstrate any significant PFS or OS benefit as compared to Cetuximab alone [39].

The mTOR pathway also provides another promising target for HNSCC—mTOR Complex 2 (mTORC2) is involved further upstream in the regulation of the cell cycle and proliferation through phosphorylation of Akt, which is regulated via the PI3K pathway as previously described. However, a meta-analysis of studies looking at mTOR inhibitors specifically demonstrated that monotherapy agents such as Temsirolimus were not able to achieve any significant tumor response—though it was able to afford some short-lived tumor control with a PFS of 1.86 months. However, this study did also demonstrate that when mTOR inhibitors were used in conjunction with chemoradiotherapy, response rates improved dramatically to 48.1%, indicating that mTOR inhibitors may be incorporated into treatment for HNSCC in combination with other agents for synergistic effect [40].

### 3.3. FGFR Alterations

Fibroblast growth factor receptor (FGFR) alterations (mutations, amplifications, fusions) occur in a subset of HNSCC [41] and play an important role in tumorigenesis. These alterations can trigger downstream activation of many signalling cascades including the PLCy and JAK/STAT pathways to cause cell growth, anti-apoptotic effects and cellular migration then invasion [42] as seen in Figure 2. FGFR inhibitors, such as erdafitinib (approved for bladder cancer with FGFR alterations), are being evaluated in clinical trials for HNSCC. A Phase 2 trial enrolling advanced/metastatic solid cancer patients with FGFR1-4 mutations or fusions demonstrated that Erdafitinib resulted in an ORR of 40% [43]. This tumor-agnostic study included a small number of HNSCC patients and provided a positive signal indicating that FGFR directed therapies have untapped potential to be explored further in the setting of HNSCC.

### 3.4. HRAS Mutations

Rat sarcoma (RAS) proto-oncogenes play an important role in the development of cancer, with the three RAS genes (HRAS, NRAS and KRAS) being the most commonly activated drivers of cancer within this family. Although RAS mutations are not commonly seen in HNSCC, it has been shown that RAS activation still occurs frequently in this cancer, acting as an on-switch to activate the RAS-RAF-MEK-ERK cascade which results in increased cell proliferation, survival and differentiation (Figure 2) [44].

The development of RAS inhibitors has been slow and the lack in success in developing efficacious RAS-targeted agents had led many to once believe that this oncoprotein was ‘undruggable’. The absence of deep hydrophobic pockets in the RAS protein structure results in the inability of small molecules to bind to this particular target [45]. However, a better understanding of this family of genes has led to the recent breakthrough and development of drugs like Sotorasib, a KRAS G12C inhibitor, resulting in reignited interest in this area. The presence of a Cys12 mutation allows for drugs to target and irreversibly bind to KRASG12C, trapping it in a quiescent, GDP-bound state. Sotorasib is now well known for its activity in KRAS G12C mutated non-small cell lung cancers and colorectal cancer, but has yet to show efficacy in the setting of HNSCC—likely due to its overall low prevalence of 2.9% [46].

Due to these challenges, there has been growing interest in pursuing HRAS mutations as a potential target instead. Though relatively rare, they are still more prevalent in HNSCC as compared to KRAS mutations [47]. Tipifarnib, a farnesyltransferase inhibitor that inhibits HRAS processing, has shown some activity in HRAS-mutant HNSCC demonstrating a response rate of 55% in Phase 2 trials enrolling refractory/metastatic HNSCC patients after progressing on standard treatment options [48].

### 3.5. Cell Cycle Regulators

The retinoblastoma (Rb) tumor suppressor gene plays a crucial role in regulating the cell cycle as mutations of this gene results in unchecked cell proliferation. This pathway may be altered through means of cyclin-dependent kinase inhibitor 2A (CDKN2A) loss, which causes activation of the CDK4/6-Cyclin D1 complex—this phosphorylates the Rb protein to release E2F transcription factors from its Rb-E2F complex, triggering gene transcription to result in acceleration of the G1/S phase of the cell cycle [49] as demonstrated in Figure 2. Because of this, CDK4/6 inhibitors have been explored as a means to inhibit this pathway, but thus far with limited success with Abemaciclib demonstrating no objective response in patients with HNSCC who had progressed through 1st line chemotherapy with Cetuximab [50].

A possible hypothesis could be the lack of sufficient tumor control with single agent therapy—Ribociclib, another CDK4/6 inhibitor, was demonstrated to only have a cytostatic effect in HPV-negative HNSCC mouse models and, additionally, had minimal effect on HPV-positive subjects [51].

CDK4/6 inhibitors are widely approved for hormone receptor-positive breast cancer and are currently being explored in HNSCC, particularly in tumors with cell cycle dysregulation (e.g., CDKN2A loss) [52]. This supports the proposition that CDK 4/6 inhibitors are best used in combination with other drugs, which is commonly seen in breast cancer where improved outcomes have been demonstrated when used in conjunction with endocrine therapy; though utility seems to be limited when used as monotherapy.

In the setting of HNSCC, however, the optimal partner drug is still unknown. In vitro and in vivo studies have demonstrated a synergistic effect of Palbociclib with Cetuximab in HNSCC—this is postulated to arise from the ability of Cetuximab to inhibit the induction of CCND1 and EGFR activation that is usually triggered by CDK 4/6 inhibitors [53]. Subsequently, combination therapy has shown far more promising results—in a Phase II trial, Palbociclib with Cetuximab in platinum-resistant but Cetuximab-naive R/M HNSCC patients demonstrated ORR of 35% with PFS of 6.4 months and OS of 12.1 months [54].

### 3.6. DNA Damage Response

Aberrant DNA repair pathways are common in SCCHN patients and contribute to tumorigenesis, with homologous recombination deficiency (HRD) and polymorphisms in single strand breaks (SSB) contributing to the cell’s impaired ability to repair damaging DNA breaks [55]. These damaged pathways can make tumors more sensitive to certain therapies like poly ADP ribose polymerase (PARP) inhibitors as depicted in Figure 2, which have had remarkable success in some cancers associated with homologous recombinant deficiencies like breast and prostate cancer [56]. In a single arm phase 2 trial, Olaparib, a highly selective PARP inhibitor, was combined with Carboplatin and Pembrolizumab in treatment naive refractory/metastatic HNSCC patients and demonstrated ORR of up to 67% [57]. Olaparib has also been safely combined with radiotherapy and Cetuximab in early phase 1 trials for locally advanced HNSCC with 2 year OS rates approximating 72%, indicating that this promising drug may work synergistically with other modalities of treatment as well [58].

## 4. Epigenetic Modifiers

Epigenetic alterations, such as DNA methylation and histone modifications, play a significant role in HNSCC pathogenesis.

### 4.1. Histone Deacetylase (HDAC) Inhibitors

Dysequilibrium of histone acetylation has been associated with cancer pathogenesis, ref. [59] and HDACs play a key role in this process—interaction with Rb to form the HDAC–Rb repressor complex regulates genes involved in the G1/S phase of the cell cycle through deacetylation of histones, which then causes condensation of the chromatin structure and reduced expression of important tumor suppressor genes [60]. HDAC inhibitors, conversely, promote hyperacetylation of lysine residues on histones to allow relaxation of its chromatin structure, leading to activation of tumor suppressor genes to downregulate cell proliferation and trigger apoptosis as depicted in Figure 2. Clinical trials are ongoing, often in combination with other therapies. Vorinostat and Pembrolizumab were evaluated in a Phase II study including both heavily pre-treated refractory/metastatic HNSCC and salivary gland cancers. In the HNSCC cohort, 32% achieved partial response and 20% achieved stable disease with an OS of 12.6 months and PFS of 4.5 months [61].

### 4.2. DNA Methyltransferase (DNMT) Inhibitors

DNA methyltransferases (DNMTs) catalyze a reaction that allows DNA methylation, thus controlling DNA transcription rates. Alterations in DNA methylation will, thus, have downstream effects on cell differentiation and proliferation as well as apoptosis [62]. Overexpression of DNMTs has been linked with a higher likelihood of metastasis and poorer prognosis in cancers due to abnormal DNA methylation [63]. DNMT inhibitors such as azacitidine and decitabine are postulated to be able to reverse these aberrant DNA methylation patterns. In vitro and in vivo models of Cisplatin-resistant HNSCC cells have demonstrated that with Decitabine, reverse methylation allowed the modification of gene expression to restore a more Cisplatin-sensitive profile [64]. DNMT inhibitors are now being evaluted in human trials in combination with immunotherapy and other agents—a phase 1B trial recently showed that the combination of azacitidine with durvalumab and tremelimumab in anti-PD-1 refractory HNSCC patients resulted in a 2 year OS rate of 24.7% [65].

### 4.3. Enhancer of Zeste Homolog 2 (EZH2) Inhibitors

EZH2 is a histone methyltransferase frequently overexpressed in HNSCC and results in cell proliferation, migration and invasion [63]. Tazemetostat is approved for other cancers such as lymphoma, with growing interest in exploring its use in solid cancers [66]. A phase 1 trial looking at the safety and tolerability of Tazemetostat in combination with Pembrolizumab in heavily pre-treated refractory/metastatic HNSCC, revealed that 800 mg of Tazemetostat was safe and tolerable in these patients though with no appreciable objective response [67]. Currently, enrolment into a phase 2 trial is ongoing and we await the results of this trial to establish the efficacy of EZH2 inhibitors in HNSCC.

## 5. Targeting the Tumor Microenvironment (TME)

The TME is composed of stromal cells, immune cells, blood vessels, extracellular matrix and plays an important role in supporting tumor growth, migration and drug resistance.

### 5.1. Anti-Angiogenic Agents

Due to rapid cell proliferation and mitotic processes, cancer cells have a higher oxygen requirement which cannot be supported by existing normal vasculature. When oxygen demand outstrips the supply, hypoxia sets in—this signal triggers the activation of hypoxia inducible factors 1 and 2 (HIF 1 and 2) and the subsequent increased expression of vascular endothelial growth factors A–D (VEGF A–D). Increased angiogenesis then facilitates further growth of the cancer and accelerated progression of tumors [68]. BAY-87-2243, a small molecular inhibitor of HIF1, was demonstrated to have improved local tumor control when given prior to radiotherapy in mice with HNSCC xenografts [69]. Further research is currently ongoing to understand how HIF inhibitors may be used meaningfully in the control of cancer, as their poor specificity also results in multiple off-target adverse effects. The efficacy of VEGF inhibitors has been somewhat disappointing in this space as well, with Bevacizumab in combination with chemotherapy affording marginally improved ORR of 9% and PFS of 1.7 months when compared to chemotherapy alone; and no improved survival in heavily pre-treated HNSCC [70]. Oral VEGF inhibitors like Sorafenib have shown even more limited success in HNSCC, with little to no response at all in previously treated patients [71]. Evidently, more needs to be performed to understand the underlying mechanism of hypoxia better and how we can integrate hypoxia-targeting agents as a possible new approach in HNSCCN.

### 5.2. Cancer-Associated Fibroblasts (CAFs)

The main sources of CAFs arise from normal fibroblasts within the tumor microenvironment, with growth factors like transforming growth factor-beta 1 (TGF-B1) and stromal cell derived factor-1 (SDF1) facilitating this transformation. These cells do not undergo apoptosis and possess enhanced cellular migration skills, as well as cytokine signalling, which promotes angiogenesis [72]. Some emerging strategies to target CAFs include neutralisation of TGF-B1 via the use of antibodies, which has been shown in vivo to allow the reconditioning of CAF in mice [73]. Other strategies to target CAFs are currently still in preclinical and early clinical development and the translation into human trials is eagerly awaited.

### 5.3. NOTCH1 Inhibition

The NOTCH pathway ultimately determines cell fate and, thus, mutations within the Notch gene result in a loss in function and increases the cell self-renewal capacity. It also modifies the TME by promoting epithelial–mesenchymal transition, angiogenesis and cell proliferation [74]. Alterations in this pathway are present in as many as 66% of HNSCC [75] and has been associated with Cisplatin resistance as well [76]. NOTCH1 inhibition was demonstrated to reduce cancer stem-like cells and cancer self-renewal abilities in vitro and in vivo studies looking at HNSCC cell lines and, thus, provide a promising target for HNSCC management [77] though once again, most of this research remains in the early pre-clinical phase.

### 5.4. Modulating the Immune Microenvironment

In addition to checkpoint inhibitors, other approaches to modulate the immune microenvironment, such as targeting myeloid-derived suppressor cells (MDSCs) and tumor-associated macrophages (TAMs), are currently being investigated. MDSCs are abundant in the TME of HNSCC and contribute to the immunosuppressed environment, undermining the anti-cancer immunity effect [78]. Similarly, TAMs allow tumor growth by hampering the immune response by impairing T cell activation and inhibiting M1 macrophage-mediated immune responses and thus, lower TAMs infiltration rates have been shown to correlate with improved survival [79]. The binding of colony-stimulating factor-1 receptor (CSF1R) to its ligand CSF1 triggers a signally cascade that regulates differentiation, proliferation, migration and survival of these macrophages [80]. CSF1R inhibition may, therefore, allow for more controlled regulation of TAMs and keep cancer growth in check. Murine HNSCC cell lines were treated with CSF1R inhibitors (BLZ945, PLX3397), and while this resulted in apoptosis of TAMs, there was no significant tumor response. This result improved only when Cisplatin was administered to these same cell lines, causing marked tumor shrinkage with immunohistochemical evaluation demonstrating increased CD8+ T cells infiltration and an enhanced antitumor immunity effect [81]. Reduction in MDSC levels have been seen with the use of all-trans retinoic acid (a vitamin A derivative) in the setting of acute promyelocytic leukemia—this drug allowed a dramatic drop in MDSC levels thereby inducing immature myeloid cells to differentiate into mature dendritic cells, which subsequently translated into a significant improvement in overall survival [82]. However, little of this has been seen of this in the HNSCC world thus far as MDSCs remain difficult to target due to the large variety and heterogeneity of these cells and the lack of a uniform marker to allow for proper selection and targeting.

## 6. Other Innovative Approaches

### 6.1. Antibody-Drug Conjugates (ADCs)

Antibody drug conjugates (ADC) contain a monoclonal antibody attached to a cytotoxic payload and have been touted to provide a good substitute to traditional cytotoxic chemotherapy drugs in view of its ability to bind selectively to a cancer cell and provide more targeted drug delivery. Conventional chemotherapeutic drugs tend to result in significant toxicities due to lack of selectivity for specific cancer cells and ADCs were created to overcome this by allowing more targeted delivery of cytotoxic treatments.

Within the molecule, the target antigen allows for recognition of target cells whilst the antibody allows guidance for drug delivery and a linker provides a bridge between the antibody and the cytotoxic drug to control the release of the drug within the cancer cell. A bystander effect is also sometimes observed when membrane-permeable payloads are able to affect neighbouring cells regardless of the target antigen expression.

Common payloads include agents that disrupt microtubule formation such as monomethyl auristatin A (MMAE) and topoisomerase-1 inhibitors like SN-38. MMAE results in cell cycle arrest by interrupting tubulin polymerization and destabilising microtubule structures. By modifying the cancer cell’s cytoskeletal structure, it can lead to rapid cell death and demonstrates high potency amongst rapidly dividing cells especially [83]. Microtubule inhibitors have good activity in HNSCC, as demonstrated by the efficacy of Paclitaxel in these patients, thus making MMAEs an attractive choice of payload in ADCs.

SN-38 is the active metabolite of Irinotecan, a topoisomerase I inhibitor that prevents topoisomerase from initiating the repair of any damaged DNA, which then leads to cell apoptosis. This payload class conventionally consists of camptothecin derivatives, which is poorly soluble in water and is significantly less effective when compared to other payloads like anti-microtubule or DNA targeting agents. Because of this reason, this payload class has been identified to have only intermediate cytotoxic activity [84].

ADCs have recently gained traction in many other solid tumor cancers such as urothelial and breast cancers, but they have yet to be FDA approved for use in HNSCC, though multiple recent trials have demonstrated good efficacy with these agents. HNSCC remains poorly represented in many of the ADC trials for solid cancers for a major reason—namely, the disease is biologically heterogenous with diverse antigen expression, making it a challenging cancer to target.

Enfortumab Vedotin is a nectin-4 targeted ADC with an MMAE payload that has been demonstrated to have good activity in the urothelial cancer setting. Nectin-4 is a protein whose overexpression encourages tumorigenesis and by promoting angiogenesis; results in tumor cell growth, proliferation, migration. It is also found in up to 80% of HNSCC patients, with non smokers or HPV positive patients showing higher rates of expression, making it an attractive target for an ADC [85]. EV202 was a Phase II study that enrolled patients with previously treated HNSCC who had progressed beyond platinum-based chemotherapy and immunotherapy. This trial demonstrated that Enfortumab Vedotin provided an ORR of up 23.9%, median OS of 5.98 months and a modest PFS of 3.9 months [86]. Unique adverse reactions included peripheral neuropathy, rash, hyperglycemia as well as ocular toxicities.

Tisotumab Vedotin was demonstrated in innovaTV 207 to have an ORR of up to 32.5% in patients who had progressed beyond platinums and checkpoint inhibitors. Median duration of response (DOR) was considerable at 5.6 months [87]. Similarly to Enfortumab, Tisotumab carries an MMAE payload but targets tissue factor (TF) instead of nectin-4. TF, an important glycoprotein in the coagulation cascade, also contributes to cancer growth in a number of ways—TF found on tumor cell results in a fibrin coat which traps these cells within the microvasculature to facilitate hematogenous seeding; TF expression also purportedly promotes angiogenesis, increased vascularity and, thus, tumor growth [88].

Sacituzumab Govitecan—another ADC more commonly known for its activity in breast and urothelial cancers—targets trophoblast cell surface antigen (TROP-2) that is expressed in multiple cancers but mostly absent in normal tissues, making it an ideal target for cancer-directed treatments. Overexpression of Trop 2 has been shown to promote tumor growth, due to activation of the ERK/MAPK pathway, which then results in acceleration of the cell cycle, whilst driving migration and infiltration of tumor cells [89]. Sacituzumab was explored in the TROPICS 03 basket trial, which included a small number of advanced HNSCC patients who had progressed on platinum-based chemotherapy and check point inhibitors. The drug demonstrated an ORR of 16% with a DOR of 4.2 months and PFS of 4.1 months, and OS has not yet been reported [90]. Though modest, this drug appears to have some clinical activity in the setting of HNSCC. With a payload of SN-38, the side effect and toxicity profile differs greatly from Enfortumab and Tisotumab Vedotin, with diarrhea and mucositis featuring more prominently.

Pucotenlimab, a recombinant humanised PDL1 inhibitor, was combined with MRG003, an antibody drug conjugate targeting epidermal growth factor receptor (EGFR) with an MMAE payload, in a Phase I/II trial and preliminary results demonstrated a response rate of up to 60% in the previously treated HNSCC cohort. These patients had to be EGFR positive to be enrolled into this particular trial and the study is currently still ongoing [91]. As discussed above, EGFR makes for an attractive target for HNSCC due to its high expression in these cancers, however as this receptor is also expressed in normal epithelial cells, there is a need to mitigate its potential toxicities when targeted by an ADC. The initial Phase I trial evaluating the use of MRG003 demonstrated that most adverse events were Grade 1–2, although 31% experienced Grade 3 events including neutropenia, febrile neutropenia, elevated aspartate aminotransferase levels, hyponatremia and leucocytopenia [92].

### 6.2. Proteolysis Targeting Chimeras (PROTACs)

PROTACs are bifunctional molecules that bind to a target and E3 ligases, which in turn activates the ubiquitin-proteasome system to trigger the degradation and silencing of oncogenic proteins. This is a relatively new technology with potential applications in HPV-positive HNSCC, as these PROTACS can be engineered to target viral oncoproteins E6 and E7 which are associated with HPV infection. This approach appears promising, as it theoretically provides a more targeted anti-cancer effect as well as a more long-lasting and durable response [93]. Preclinical studies have demonstrated that the use of PROTAC in HNSCC cell lines have some anti-tumor effect in causing the loss of colony-forming abilities by degrading leucine zipper-bearing kinase (LZK), a protein that encourages HNSCC growth and proliferation [94]. However, as a new and novel strategy, further research is still needed to assess the efficacy of PROTACs in HNSCC human models.

### 6.3. Metformin

Metformin, a biguanide, is better known for its hypoglycemic effects in the world of diabetes. However, it may potentially provide anti-cancer control as it triggers AMP protein kinase (AMPK) related pathways leading to inactivation of MTOR which subsequently leads to suppression of its downstream signalling effects [95]. Early clinical data suggests that this may translate into anti-cancer and tumor growth inhibitive effects—Metformin was administered to HNSCC patients and flow cyometry results taken before and after administration subsequently demonstrated expanded natural killer cell populations, increased anti-tumorigenic cytokine profiles and a change in CD8+ T cell memory phenotypes [96]. This suggests that Metformin has an immunomodulating effect and with its favourable side-effect profile, this drug appears to yield significant potential to be used as an adjunctive therapy in HNSCC patients.

### 6.4. Radiopharmaceuticals

Targeted radioligand therapy (TRT), which allows delivery of DNA-damaging radioactive isotopes to tumor cells with specific target molecules, is being explored. EGFR again, provides much promise as a potential target, with preclinical HNSCC models demonstrating that radiolabelled Panitumumab and Cetuximab—both anti-EGFR monoclonal antibodies—exhibited good tumor targeting efficacy with some tumor growth inhibition [97]. This early data suggests that TRTs may be a new class of drugs that can potentially be integrated into the treatment landscape of HNSCC in the future.

**Figure 3:**
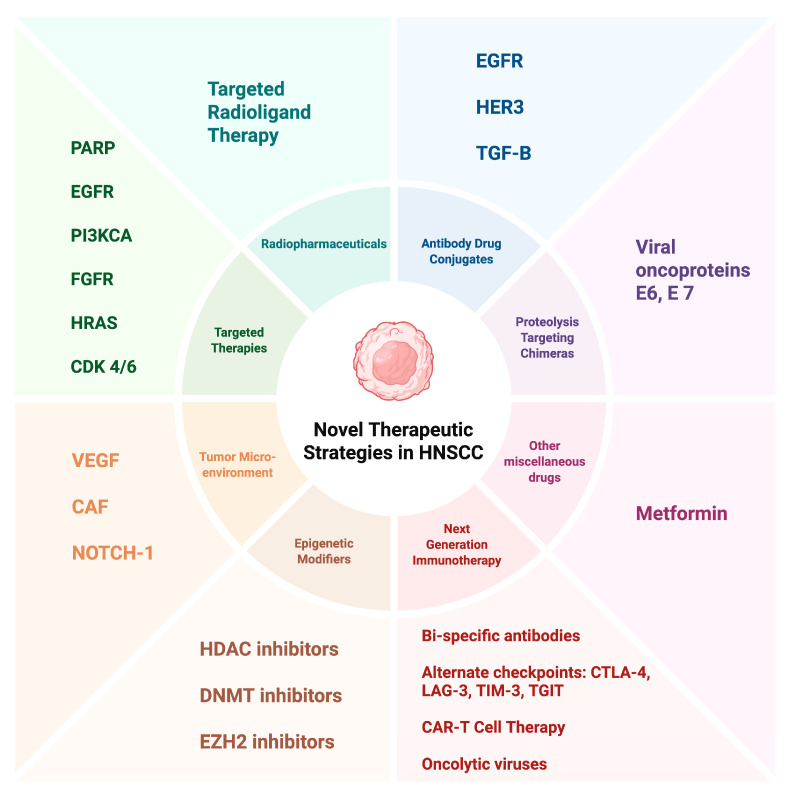
A summary of the different subtypes and classes of novel treatments that are emerging in the treatment of HNSCC. CAR, chimeric antigen receptor; CAF, cancer-associated fibroblasts; CDK, cyclin-dependent kinase; CTLA-4, Cytotoxic T lymphocyte-associated antigen 4; DNMT, DNA methyltransferase; EGFR, epidermal growth factor receptor; EZH2, enhancer of Zeste Homolog 2; HER3, human epidermal growth factor receptor 3; FGFR, fibroblast growth factor receptor; HDAC, histone deacetylase; HNSCC, head and neck squamous cell carcinoma; HRAS, Harvey rat sarcoma viral oncogene; LAG-3, Lymphocyte-activation gene 3; PARP, poly ADP ribose polymerase; PI3KCA, Phosphatidylinositol 3-kinase catalytic subunit alpha; PDL-1, programmed death-ligand 1; TGF-B, transforming growth factor-beta; TGIT, T cell immunoreceptor with Ig and ITIM domains; TIM-3, T-cell immunoglobulin and mucin-domain containing-3; VEGF, vascular endothelial growth factor.

In conclusion, the face of HNSCC treatment is changing rapidly with many new agents and treatment options on the horizon. Beyond conventional chemotherapy and checkpoint inhibitors, we are now moving closer towards precision oncology so as to improve treatment effectiveness, minimize side effects, and ultimately improve patient outcomes. Figure 3 summarizes the different approaches wherein we can attempt to fine-tune our treatments according to tumor molecular characteristics and biology.

Table 1 provides a concise summary of how some of these methods have been implanted in the recent trials cited within this review article. Though many of these studies are still within the early phase, they still provide an encouraging signal with respect to the efficacy of alternative means of treatments to refractory or relapsed/metastatic HNSCC.

## 7. Challenges and Future Directions

**Figure 4:**
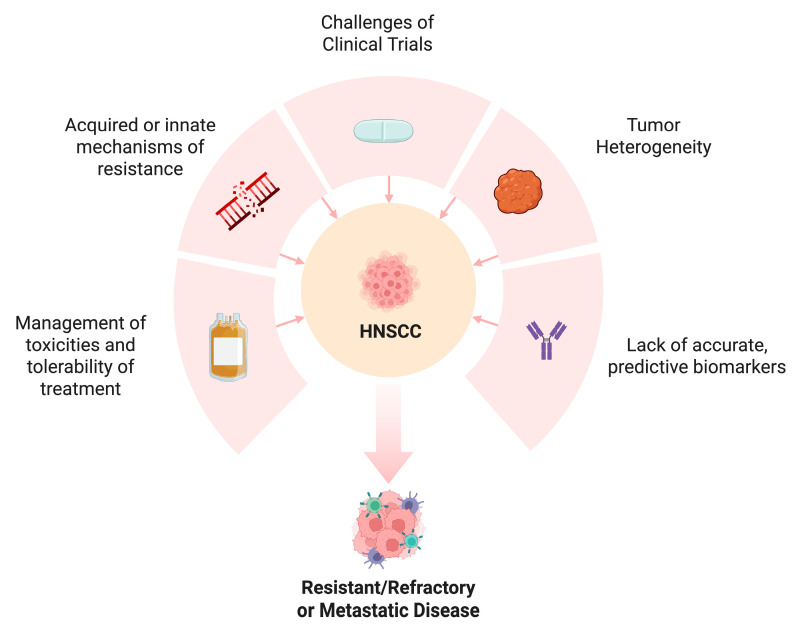
The challenges of treating resistant, refractory or metastatic HNSCC.

In spite of many new alternative approaches and emerging strategies, HNSCC remains a challenge to treat due to many reasons. Figure 4 depicts many of these barriers and obstacles impeding on the advancement of our treatments of HNSCC.

### 7.1. Overcoming Resistance

Identifying and targeting mechanisms of resistance to both targeted therapies and immunotherapy is a major challenge. Acquired resistance may arise after exposure to Cetuximab, possibly through activation of the RAS/RAF/MEK/ERK pathway which results in phosphorylation of proteins involved in cell proliferation, differentiation and anti-apoptosis activity [22]. Cisplatin resistance has been postulated to arise from microRNAs that induce Twist expression through signalling via c-Jun N-terminal kinase activity, a downstream target of the MAPK pathway, resulting in inhibition of cisplatin-induced apoptosis [98]. Increased drug transport of Cisplatin may also contribute to an adaptive resistance—increased expression of ATP-binding cassette (ABC) transporters due to activation of the Hedgehog signaling pathway have been implicated in acquired chemoresistance to 5-fluorouracil and Cisplatin in SCCHN cell lines [99]. Resistance to Cisplatin also arises when adaptive responses allow improved DNA repair mechanisms in response to Cisplatin-induced damage, thereby abolishing its cytotoxic effects [100]. It is evident that the mechanisms of Cisplatin resistance in pre-treated HNSCC can be rather complex and multi-faceted, hence combination therapies are likely to be crucial.

### 7.2. Biomarker Development

Current established biomarkers for predicting the response of HNSCC cancers to treatment include PD-L1, HPV positivity, tumor mutational burden and microsatellite instability. However, the true predictive value of these biomarkers has often been questioned. For example, currently there are discordant results across many studies with regard to the role of PD-L1 and selecting patients who may benefit from immunotherapy. This is partially due to the lack of uniformity between assays as well as the different thresholds used by different trials to determine PD-L1 positivity. Furthermore, PD-L1 expression is regulated by other signalling pathways as well, such as MAPK and AKT—hence, its expression may change over time from the point of initial diagnosis through progression of disease [101]. More reliable biomarkers are needed to predict response to specific therapies and guide treatment decisions.

### 7.3. Addressing Tumor Heterogeneity

Strategies to address both intratumoral and intertumoral heterogeneity are needed. Though most HNSCCs originate from the squamous cell, each subtype of this cancer has its own unique TME, contributing to each tumour’s heterogeneity. The clearest example exists between viral- vs. non-viral-related HNSCC—HPV-related oropharyngeal cancers have a better treatment outcome and prognosis as compared to their HPV-negative counterparts [102]. This is likely related to the fact that HPV driven cancers tend to have better chemosensitivity. Additionally, as these cancers also tend to have a more inflammatory tumour microenvironment with increased cytokine production, this contributes to increased sensitivity to radiotherapy [103]. Many of these patients also tend to be younger, non-smokers and have minimal co-morbidities, which contribute to better tolerance of treatment and higher treatment completion rates [104]. These two subsets of oropharyngeal cancers also differ in their underlying genetic alterations—HPV-positive HNSCCs are characterized by higher number of chromosomal alterations and amplifications as well as different genetic expression profiles [105].

Emerging data suggests that we may even be able to further risk-stratify HPV related oropharyngeal cancers with functional imaging to offer de-escalation of radiotherapy treatments. In the 30 ROC trial, patients with no tissue hypoxia at baseline positron emission tomography (PET) imaging were treated with 30 Gy of radiotherapy, demonstrating similar outcomes as their counterparts with significant baseline tissue hypoxia who received 70 Gy [106]. Tumor heterogeneity exists even within the same subclass of HNSCCs, demonstrating that biology-driven approaches are important to consider when tailoring treatments, as compared to empiric approaches.

Nasopharyngeal cancer (NPC) provides another example with regards to the heterogeneity of HNSCC. Though it arises from the head and neck region, its risk factors are drastically different—with Epstein–Barr Virus (EBV) infection and ancestry (originating from Southeast Asia, China, North Africa, Middle East) being the major perpetrators of this disease [107]. Due to challenging anatomy with close proximity to major neurovascular structures as well as its remarkable sensitivity to radiotherapy, NPC is commonly treated with combination chemoradiotherapy as opposed to the surgical modalities that are usually employed in HNSCC.

While the subtypes of HNSCC are clinically and biologically different, the lack of targeted treatments customized for each subset of this cancer has resulted in these diseases being categorised into a single entity and, thus, being treated in the same way. Unsurprisingly, outcomes have remained disappointing and the rates of poor survival highlight the need for more personalized treatment options and new approaches to tackle this challenging group of cancers and its varied tumor heterogeneity.

### 7.4. Clinical Trial Design

Innovative trial designs, including adaptive trials and platform trials, are needed to efficiently evaluate novel therapies in HNSCC. However, there still remain many barriers to conducting good clinical trials in HNSCC, such as insufficient accrual due to its overall lower incidence compared to the other more common cancers. The diversity of treatment standards across different countries as well as fewer therapeutic options available also adds to this challenge.

### 7.5. Toxicity Management

Careful attention to potential toxicities is crucial—while many of these treatments are efficacious, they are associated with significant toxicities. This is especially relevant to our HNSCC patients—due to the location and morbidity of these cancers, patients tend to be at higher risk of oral health complications which can quickly lead to malnutrition and also negatively affect their quality of life. Gastrointestinal adverse effects of systemic therapies can further exacerbate this, placing these patients at additional nutritional risk and diminished function.

## 8. Conclusions

Drug development in HNSCC is rapidly evolving, with a focus on novel therapeutic strategies that go beyond traditional approaches. Figure 3 depicts these new approaches in the form of next-generation immunotherapies, targeted therapies directed at specific molecular alterations, epigenetic modifiers, and agents targeting the TME that hold promise for improving outcomes for patients with this challenging disease. Continued research, collaboration, and innovative clinical trial designs are essential to translate these promising preclinical findings into clinical benefit. The promise of new therapeutic options have reignited interest and enthusiasm in a field that has been otherwise stagnant, and the results of larger and randomized trials using these approaches are eagerly awaited.

## Figures and Tables

**Table 1:** A review of the latest trials involving current novel approaches in refractory or metastatic HNSCC.

Trial Reference No.	Phase	N	Cohort	Investigational Drug	ORR (%)	PFS (Months)	DCR (%)
NCT03005782	I	30	R/M HNSCC 2 cohorts: Anti–PD-1/PD-L1-naïve (Cohort 11) or anti–PD-1/L1-experienced (Cohort 12)	Fianlimab (LAG-3 inhibitor) and Cemiplimab	33% (Cohort 11) 7% (Cohort 12)	2 (Cohort 11) 4 (Cohort 12)	47% (Cohort 11) 67% (Cohort 12)
NCT03625323	II	39	R/M HNSCC who had failed first-line platinum-based therapy, unselected for PD-L1	Eftilagimod Alpha (LAG-3 inhibitor) with Pembrolizumab	29.7%	2.1	Not reported
NCT04429542	I/Ib	39	R/M HNSCC with a tumor PD-L1 CPS ≥ 1 with no prior systemic therapy	BCA 101 (bispecific antibody targeting TGF-B and EGFR) and Pembrolizumab	46%	Not reached	Not reported
NCT03526835	II	26	R/M HNSCC with no prior systemic therapy, PDL1 positive	Petosemtamab (bispecific antibody targeting EGFR and LGR-5) and Pembrolizumab	67%	Not reported	Not reported
NCT05054439	I/Ib	29	R/M HNSCC progressed on prior anti-PD-1/L1 with or without platinum-based chemotherapy and received no more than two lines of treatment	Group A, pts without prior exposure to paclitaxel - Izalontamab (bispecific antibody targeting EGFR and HER2) with Paclitaxel Group B, pts with prior exposure to paclitaxel - Izalontamab with Docetaxel	Group A: 64.3% Group B: 12.5%	Group A: 5.6 Group B: 1.9	Group A: 92.9% Group B: 62.5%
NCT03381183	Ib	16	R/M HNSCC, may or may not have had PD1 inhibitor therapy before	IRX2 and Durvalumab	5.3%	6.18	42%
EudraCT2005-000777-21	I/II	17	Stage III/IVA/IVB HNSCC	Intratumoral injections of oncolytic herpes simplex type-1 virus (HSV-1) encoding human granulocyte-macrophage colony-stimulating factor (GM-CSF), with chemoradiotherapy followed by surgical resection	82.3%	Not reported	Not reported
NCT04083976	II	178 (10 HNSCC)	Advanced or metastatic solid tumors of any histology with predefined FGFR1-4 alterations, progressed on 1 or more lines of systemic therapy	Erdafitinib	40%	5.2	Not reported
NCT02383927	II	30	R/M HNSCC patients with ≥1 prior platinum-containing regimen	Tipifarnib (farnesyltransferase inhibitor that disrupts HRAS function)	55%	5.6	Not reported
NCT02101034	II	30	Platinum-resistant, cetuximab-naive HPV (−) R/M HNSCC	Palbociclib and Cetuximab	35%	6.4	Not reported
NCT04643379	II	12	R/M HNSCC with no prior treatment	Olaparib, Carboplatin and Pembrolizumab	67%	Not reported	Not reported
NCT02538510	II	25	Incurable HNSCC progressed on standard therapy but no prior immunotherapy	Vorinostat and Pembrolizumab	32%	4.5	Not reported
NCT03019003	Ib	12	R/M HNSCC who progressed on anti-programmed cell death protein PD-1 (anti-PD-1) therapy	Azacitidine, Durvalumab and Tremelimumab	Not reported	Not reported	Not reported
NCT04624113	I-II	12	R/M HNSCC with PD-L1 positive tumors, progressed through standard therapies	Tazometostat with Pembrolizumab	0%	2.1	41%
NCT04225117	II	46	R/M HNSCC progressed through platinum-based chemotherapy and PD-1/PD-L1 inhibitor therapy, with ≤2 previous lines of cytotoxic therapy	Enfortumab Vedotin (antibody drug conjugate targeting Nectin-4)	23.9%	3.9	56.5%
NCT03485209	II	40	R/M HNSCC progressed through platinum based chemotherapy and checkpoint inhibitors	Tisotumab Vedotin (antibody drug conjugate targeting tissue factor)	32.5%	4.2	Not reported
NCT03964727	II	43	Locally advanced or metastatic HNSCC that progressed following platinum-based chemotherapy and anti-PD-(L)1 therapy	Sacituzumab Govitecan (antibody drug conjugate targeting Trop 2)	16%	4.1	Not reported
NCT05688605	I/II	33 (6 HNSCC)	EGFR-positive patients with refractory advanced squamous cell carcinomas of the head and neck (HNSCC)	MRG003 (antibody drug conjugate targeting EGFR) with Pucotenlimab	60%	Not reported	Not reported

Abbreviations: R/M: recurrent/metastatic; ORR: objective response rate; PFS: progression-free survival; DCR: disease control rates.

## Abbreviations

The following abbreviations are used in this manuscript:

ADC	Antibody drug conjugate
CAF	Cancer-Associated Fibroblasts
CAR	Chimeric-antigen receptor
CCND1	Cyclin D1
CDK 4/6	Cyclin-Dependent Kinase 4/6
CDKN2A	Cyclin-dependent kinase inhibitor 2A
CPOS	Combined positive score
CTLA	Cytotoxic T lymphocyte-associated antigen
DOR	Duration of response
DNMT	DNA methyltransferase
EBV	Epstein–Barr Virus
EGFR	Epidermal growth factor receptor
ERK	Extracellular Signal-Regulated Kinase
EZH2	Enhancer of Zeste Homolog 2
FDA	Food and Drug Administration
FGFR2	Fibroblast growth factor receptor
HDAC	Histone deacetylase
HER3	Human epidermal growth factor receptor 3
HIF	Hypoxia inducible factor
HPV	Human papillomavirus
HRAS	Harvey rat sarcoma viral oncogene homolog
HRD	Homologous repair deficiency
HSV	Herpes simplex virus
IL	Interleukin
LAG-3	Lymphocyte-activation gene 3
LGR-5	Leucine-rick repeat-containing G-protein
MAPK	Mitogen-activated protein kinase
MDSC	Myeloid-derived suppressor cells
MMAE	Monomethyl auristatin A
MEK	Mitogen-activated protein kinase
mTOR	Mammalian target of rapamycin
LZK	Leucine zipper-bearing kinase
NPC	Nasopharyngeal cancer
ORR	Objective response rate
OS	Overall survival
PARP	Poly ADP ribose polymerase
PFS	Progression free survival
PI3KCA	Phosphatidylinositol 3-kinase catalytic subunit alpha
RAF	Rapidly accelerated fibrosarcoma
RAS	Rat sarcoma
Rb	Retinoblastoma
R/M	Recurrent or metastatic
HNSCC	Head and neck squamous cell carcinoma
SDF1	Stromal cell derived factor-1
SSB	Single strand breaks
S6K	Ribosomal S6 kinase
TAM	Tumor associated macrophage
TF	Tissue factor
TGF-B	Transforming growth factor-beta
TGIT	T cell immunoreceptor with Ig and ITIM domains
Trop	Trophoblast cell surface antigen
TRT	Targeted radioligand therapy
TIM-3	T-cell immunoglobulin and mucin-domain containing-3
TKI	Tyrosine kinase inhibitor
TME	Tumor microenvionment
VEGF	Vascular endothelial growth factor
4E-BP1	Eukaryotic translation initiation factor 4E binding protein 1
